# Preparation of Barium Europium Phosphate and Its Performance in Acrylic Resin Anti-Corrosion Coating

**DOI:** 10.3390/polym17141966

**Published:** 2025-07-17

**Authors:** Xuying Deng, Jihu Wang, Shaoguo Wen, Jiale Zhao, Xue Zhang, Yicheng Zhao, Zhiying Deng

**Affiliations:** College of Chemistry and Chemical Engineering, Shanghai University of Engineering Science, Shanghai 201620, China; 18249632024@163.com (X.D.); wangjihu@163.com (J.W.); jasent07@163.com (J.Z.); zhangxue000110@163.com (X.Z.); 13916621852@163.com (Y.Z.); q1435445068@163.com (Z.D.)

**Keywords:** high-temperature solid-phase method, barium europium phosphate, filler, electrochemistry, anti-corrosion

## Abstract

Acrylic resin is a polymer with strong crosslinking density and strength, and it is commonly used as a matrix in water-based coatings. Barium europium phosphate (Ba_3_Eu(PO_4_)_3_) is a novel functional filler that is expected to provide anti-corrosive effects to coatings. In this study, Ba_3_Eu(PO_4_)_3_ was prepared by the high-temperature solid-phase method and applied to acrylic anti-corrosion coatings. The influence of the molar ratio of reactants on Ba_3_Eu(PO_4_)_3_ purity was studied. The anti-corrosion performance of the coating was investigated. It was found that, when BaCO_3_:Eu_2_O_3_:(NH_4_)H_2_PO_4_ = 3:0.5:3 and the reaction was carried out at 950 °C for 1000 min, high-purity Ba_3_Eu(PO_4_)_3_ can be obtained, according to XRD and EDS tests. SEM shows that Ba_3_Eu(PO_4_)_3_ has good crystal morphology and a porous morphology. TEM revealed that its structure was intact. When Ba_3_Eu(PO_4_)_3_ was added to a relative resin content of 5 wt%, the anti-corrosion performance of the coating was the best after 168 h, with the lowest Tafel current density of 9.616 μA/cm^2^ and the largest capacitance arc curvature radius. The salt spray resistance test showed that the corrosion resistance of the 5 wt% Ba_3_Eu(PO_4_)_3_ coating was also the best, which is consistent with the results of the electrochemical test. Ba_3_Eu(PO_4_)_3_ as a pigment and filler can effectively improve the anti-corrosion performance of water-based industrial coatings.

## 1. Introduction

In recent years, with the rapid development of many fields, such as construction, transportation, petrochemicals, and hydropower, anti-corrosion coatings have entered a golden period of development. In 2023, the production of and demand for anti-corrosion coatings in China reached 8.913 million tons and 8.707 million tons, respectively. These coatings can be used to prevent and reduce huge economic losses and disasters caused by corrosion problems [1]. Coatings are a very economical and effective method of corrosion protection [2,3]. The interfacial adhesion and material composition of coatings are important indicators for the improvement of their corrosion reduction performance. According to the “14th Five Year Plan for China’s Coatings Industry” released by the China Coatings Industry Association [4], in order to protect the environment, it is recommended to use water-based resins with extremely low VOC content and to discard heavy metals, such as lead and chromium, from the fillers. Instead, fillers with functional or structural characteristics should be used to slow down the corrosion rate of coatings. The problem of corrosion control still needs to be addressed further [5,6,7,8].

Rare-earth phosphates are considered effective corrosion inhibitors for protecting metals and have been used to suppress the corrosion of different metal alloys [9]. The main reason is that rare-earth phosphates can not only refine the grain size on the surface of the substrate but can also improve the adhesion between the coating and the metal substrate, reduce the porosity of the coating, and thus enhance the corrosion resistance of the coating. Highly reactive trivalent rare-earth metal ions react with the matrix resin to obtain a substance with extremely strong bond energy, thereby further improving the corrosion and aging resistance of the substrate [10]. In addition to rare-earth cerium (Ce), there have also been many studies on rare-earth europium (Eu) in anti-corrosion applications. Li [11] modified the surface of organic compounds using the structural characteristics of vanadium oxide, neodymium oxide, and hexahydrate europium nitrate. The coating, prepared by mixing with nano-modified acetylene black material, had a continuous hydrophobic structure and strong adsorption ability, which can solve the problems of corrosion and detachment of rare-earth magnesium silicon aluminum surfaces. Amir [12] combined Eu_2_O_3_ with epoxy resin and found that the corrosion resistance of scratch coatings to saltwater erosion was significantly improved by about 57%. Therefore, the application of rare-earth europium in anti-corrosion coatings needs further investigation.

Ba_3_Eu(PO_4_)_3_ serves as an anti-corrosion filler for two primary reasons. Firstly, it introduces PO_4_^3−^ ions, which enhance the adhesion between the coating film and the substrate. Secondly, it incorporates rare-earth Eu^3+^ ions. These ions exhibit characteristic red (or other) warning colors under different UV excitation wavelengths. This luminescent behavior offers a theoretical basis for current research into self-warning and self-healing coatings. This article investigates the effects of different reaction ratios on the purity of Ba_3_Eu(PO_4_)_3_ synthesized by the high-temperature solid-phase method. The purity and elemental distribution of Ba_3_Eu(PO_4_)_3_ were analyzed through XRD and EDS. The microstructure and crystal structure were tested using SEM and TEM, and the pore diameter was calculated. Finally, the effects of different amounts of Ba_3_Eu(PO_4_)_3_ on the anti-corrosion performance of acrylic coatings were investigated using an electrochemical workstation and a salt spray machine.

## 2. Materials and Methods

### 2.1. Materials

Barium carbonate (BaCO_3_, AR) was purchased from Tianjin Jinhui Taiya Chemical Reagent Co., Ltd. (Tianjin, China). Europium oxide (Eu_2_O_3_, AR) and erbium oxide (Er_2_O_3_, AR) were purchased from Nanjing Chemical Reagent Co., Ltd. (Nanjing, China). Ammonium dihydrogen phosphate (NH_4_)H_2_PO_4_, AR) was purchased from Suzhou Furun Huagong Technology Co., Ltd. (Suzhou, China). Anhydrous ethanol was purchased from the National Pharmaceutical Chemical Reagent Co., Ltd. (Shanghai, China). Acrylic resin (industrial grade) came from Shanghai Qixiang Qingchen New Material Technology Co., Ltd. (Shanghai, China). The pH regulator (DMAE, industrial grade) was purchased from Hunan Youxin Material Technology Co., Ltd. (Hunan, China). The film-forming agent (alcohol ester 12, industrial grade) was purchased from Shandong Xinheng Chemical Co., Ltd. (Jinan, China). The anti-flash-rust agent (30% NaNO_2_, LR) was self-made in the laboratory. Dispersant 5040 (sodium polycarboxylate), defoamer C-15, and wetting agent G-033 were all industrial grade and came from Anhui Guangcheng New Materials Co., Ltd. (Chuzhou, China). HTK-3020 leveling agent (industrial grade) was purchased from Shanghai Hongtu Industrial Co., Ltd. (Shanghai, China). Ethylene glycol butyl ether (AR) was provided by Shanghai McLean Biochemical Co., Ltd. (Shanghai, China). Thickener WT-105A (industrial grade) was purchased from Shanghai Kaiyin Chemical Co., Ltd. (Shanghai, China). Distilled water was self-made in the laboratory. All of the above reagents were used as received.

### 2.2. Preparation of Ba_3_Eu(PO_4_)_3_

Using the high-temperature solid-phase method, BaCO_3_, Eu_2_O_3_, and (NH_4_)H_2_PO_4_ were weighed in a certain stoichiometric ratio (with mole ratios of 3:1:3, 3:0.7:3, and 3:0.5:3, respectively) and ground in an agate mortar for 30 min. The mixture was collected in a corundum crucible and transferred to a muffle furnace. It was preheated at high temperature for 1000 min at 950 °C, cooled to room temperature, crushed, ground, and sieved. It was calcined again at 950 °C for 1000 min, cooled down, and ground to obtain a white solid powder, which was the europium barium phosphate filler.

### 2.3. Preparation of Anti-Corrosion Coating

According to the formula in Table 1, DI water, DMAE, dispersant 5040, and defoamer were added into the material cylinder and stirred at 300 rpm for 10 min and the pH value was adjusted to around 8.5. Then, different amounts of Ba_3_Eu(PO_4_)_3_ (0 wt%, 1 wt%, 2 wt%, 3 wt%, 5 wt%) were mixed with DI water for 30 min before adding them to the drum. The speed was increased to 1000 rpm and stirred for 60 min. Subsequently, the rotating speed was reduced to 500 rpm and acrylic acid lotion and other additives were added and dispersed evenly for 15 min. Finally, ethylene glycol butyl ether, DI water, and WT-105A were mixed in a ratio of 1:1:1 to obtain the coating.

The tinplate was polished with 600-mesh sandpaper, wiped with deionized water, washed with absolute ethanol, and dried for use. The coating was applied to the tinplate using a wire rod and dried to form a film. The coating thickness was 100 ± 2 μm.

### 2.4. Characterization

The BRAGG110 X-ray diffractometer (Bruker, Karlsruhe, Germany) was used to analyze the crystal structure and purity of europium barium phosphate prepared by different molar ratios of barium, europium, and phosphorus. The testing conditions were K_α_-rays from a Cu target, with a wavelength of 1.5405 nm. The scanning range and speed were 5–80° and 0.02°/s.

The microstructure of europium barium phosphate was observed using a ZEISS Gemini 300 scanning electron microscope (SEM) (ZEISS, Shanghai, China) and EDS/mapping spectroscopy was performed based on it to analyze the distribution of europium barium phosphate elements.

The morphology of europium barium phosphate was observed by using JEOL-2100F transmission electron microscope (TEM) (JEOL, Tokyo, Japan).

The corrosion status of the coating was evaluated by using CHI660E electrochemical workstation (CH Instruments, Inc., Huntington Beach, CA, USA). The electrochemical impedance spectroscopy (EIS) and Tafel polarization curve (Tafel) were plotted to analyze the corrosion resistance performance of the coatings. A 3.5 wt% NaCl solution was used as the electrolyte solution at room temperature.

The tinplate coating samples were placed in the CHY-7B salt spray test chamber for anti-corrosion testing for different resistance times according to ASTM-B117 [13]. The salt spray environment was 3.5 wt% NaCl solution.

## 3. Results and Discussion

### 3.1. XRD of Ba_3_Eu(PO_4_)_3_

**Figure 1:**
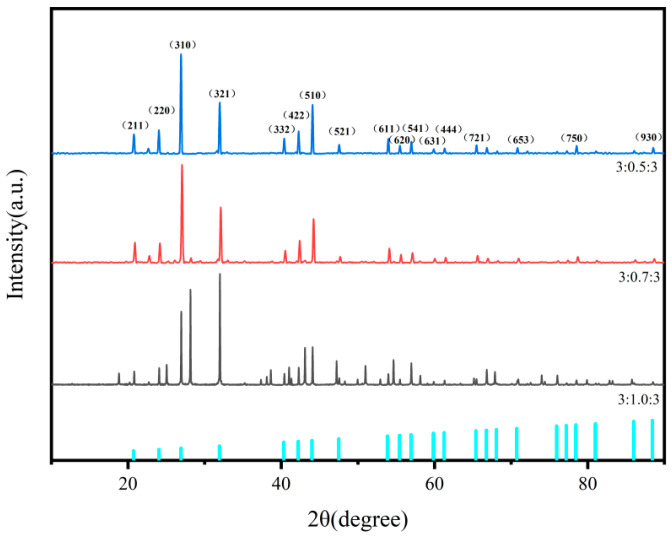
XRD patterns of Ba_3_Eu(PO_4_)_3_ with different ratios of BaCO_3_, Eu_2_O_3_, and (NH_4_)H_2_PO_4_.

Ba_3_Eu(PO_4_)_3_ was prepared by selecting BaCO_3_:Eu_2_O_3_:(NH_4_)H_2_PO_4_ in different molar ratios of 3:1:3, 3:0.7:3, and 3:0.5:3, respectively. The crystal phase structure was analyzed by XRD, and the experimental results are shown in Figure 1.

The standard peaks were simulated through software, and the peaks of the synthesized products with different molar ratios were compared. When the molar ratio of the substance was 3:1:3, there were more impurities, such as Ba_3_(PO_4_)_2_ and Eu_2_O_3_, which may indicate insufficient reaction. When the molar ratio of the substance was 3:0.7:3, there was still a small peak of Ba_3_(PO_4_)_2_. When the molar ratio of the substance was 3:0.5:3, there was a peak which was consistent with the standard peak. Compared with the standard PDF card of barium phosphate (25-0028), there was no Ba_3_(PO_4_)_2_ and Eu_2_O_3_ impurity peak at the crystal planes (111), (200), (220), and (311) [14]. This proved that the reaction was complete. The strong and sharp diffraction peaks indicated good crystallinity. The sample belongs to the monoclinic crystal system and matches with standard card JCPDS No. 29-0162.

The Scherrer equation D = kλ/β cosθ was used to calculate the crystallite size. λ was the X-ray wavelength, approximately 0.1541 nm (Cu K_α_ radiation). k was the shape factor. For amorphous crystals, the literature commonly assigns it a value of 0.89 [15,16,17]. β was the full width at half maximum (FWHM) of the sample’s diffraction peak, which needs to be converted into radians during the calculation process. The strongest peak at 2θ = 31.924° was used, and its β value was obtained from the analysis software. Among them, the FWHM was 0.089°, and the unit converted β value was about 0.0016. The calculated average crystallite size (D) was approximately 89.09 nm.

Based on the investigation of reactant molar ratios discussed in Figure 1, the optimal molar ratio for synthesizing pure Ba_3_Eu(PO_4_)_3_ was determined to be 3:0.5:3. Considering the thermal decomposition temperatures of the reactants, 950 °C was selected for calcination.

**Figure 2:**
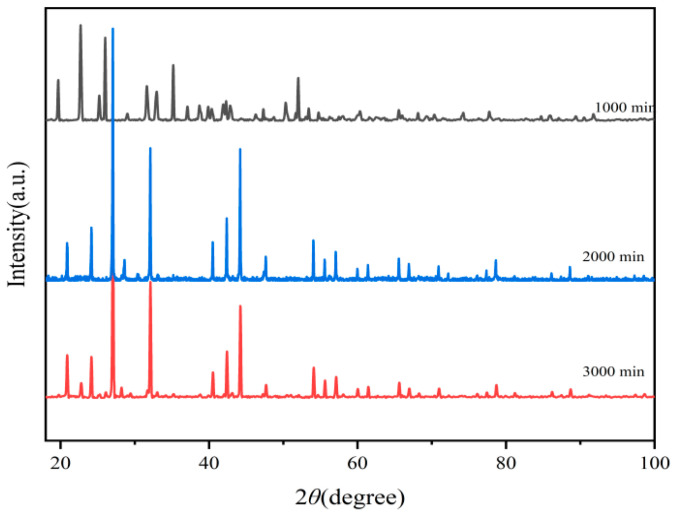
XRD patterns of Ba_3_Eu(PO_4_)_3_ generated within different reaction times.

Figure 2 showed that the calcination time of 1000 min was strongly insufficient, and the majority of the product was Ba_3_(PO_4_)_2_. In contrast, the products calcined for 2000 min and 3000 min matched well with the standard diffraction peaks according to JCPDS No. 29-0162.

### 3.2. EDS of Ba_3_Eu(PO_4_)_3_

**Figure 3:**
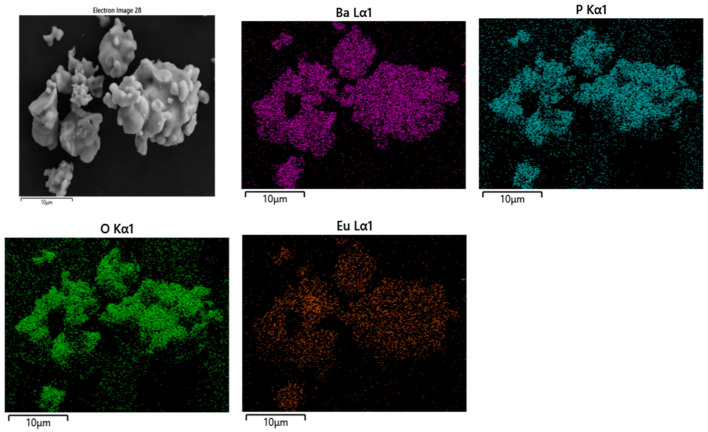
SEM spectrum and elemental mapping image of Ba_3_Eu(PO_4_)_3_.

**Figure 4:**
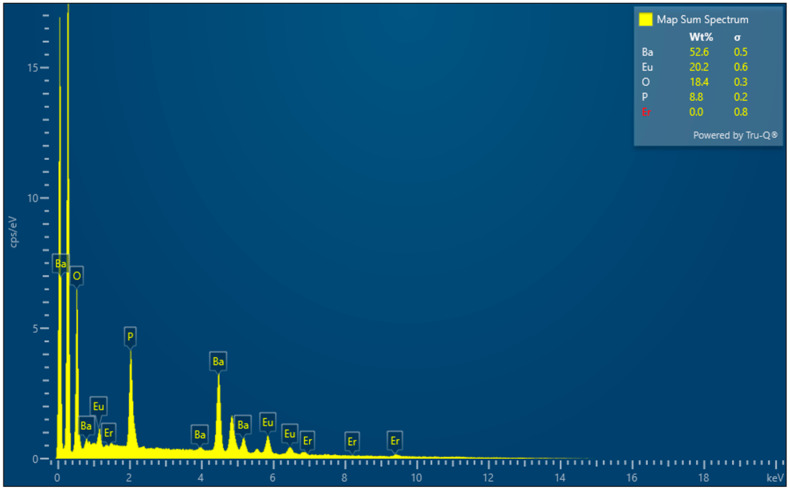
EDS image of Ba_3_Eu(PO_4_)_3_.

The elemental mapping images and EDS spectra of Ba_3_Eu(PO_4_)_3_ samples prepared with a molar ratio of 3:0.5:3 were analyzed by SEM testing. The experimental results are shown in Figure 3 and Figure 4.

From Figure 3, it can be seen that the surface of Ba_3_Eu(PO_4_)_3_ fluorescent powder particles is smooth, and Ba, Eu, P, and O elements were uniformly distributed in them.

Analyzing the EDS image of Ba_3_Eu(PO_4_)_3_ in Figure 4, it can be found that the Ba, Eu, and P mass fractions were 52.6%, 20.2%, and 8.8%. It was confirmed that the mole ratio of the substance was about 3:0.5:3. Eu_2_O_3_ reacted completely with BaCO_3_ and (NH_4_)H_2_PO_4_, which converted into Ba_3_Eu(PO_4_)_3_. The appearance of trace Er elements may be due to impurities in the raw materials.

### 3.3. Morphology of Ba_3_Eu(PO_4_)_3_

**Figure 5:**
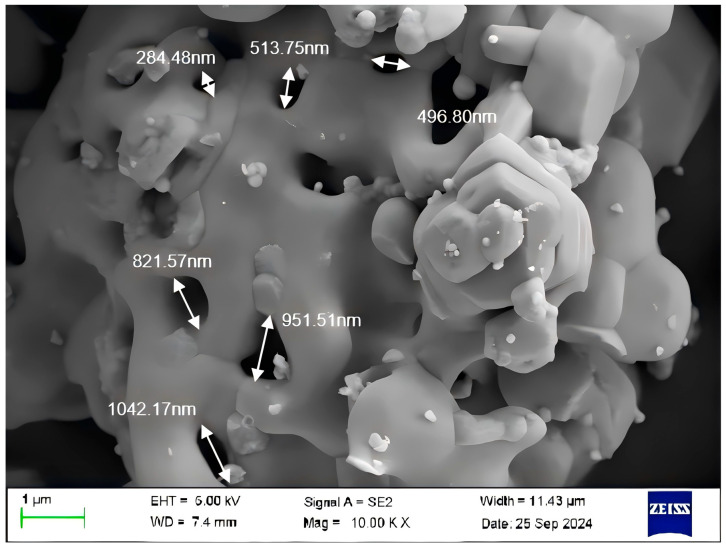
SEM image of Ba_3_Eu(PO_4_)_3_.

SEM was performed on Ba_3_Eu(PO_4_)_3_ prepared at the molar ratio of 3:0.5:3. The result is shown in Figure 5.

From Figure 5, it can be seen that the shape of Ba_3_Eu(PO_4_)_3_ was similar to a honeycomb-like pore structure, with pore diameters ranging from 284.48 to 1042.17 nm. Ba_3_Eu(PO_4_)_3_ synthesized by a high-temperature solid-phase method has the characteristics of a large specific surface area and high porosity, but there was an aggregation phenomenon. The main reason was the impact force released by a large amount of hot gases (CO_2_, NH_3_) during the reaction synthesis at high temperatures, resulting in the formation of more pores with irregular shapes.

**Figure 6:**
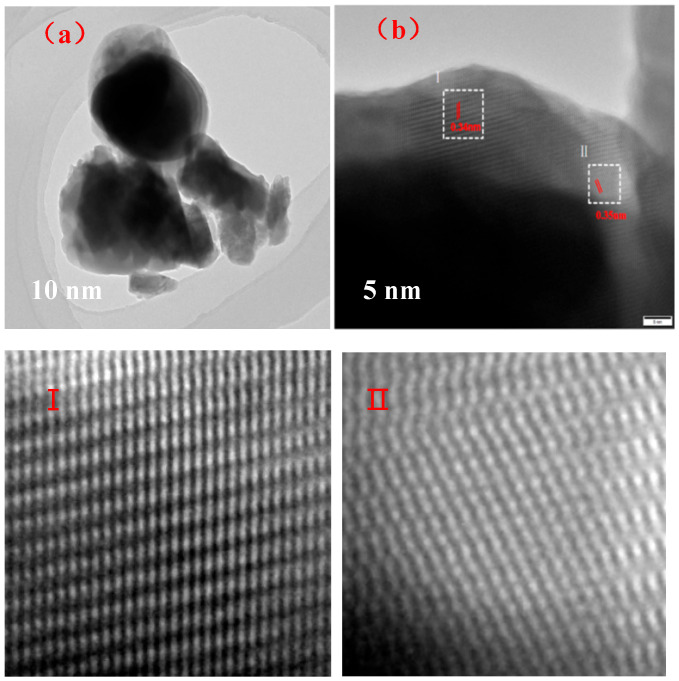
TEM image of Ba_3_Eu(PO_4_)_3_ at different magnifications: (**a**) 10 nm; (**b**) 5 nm.

Ba_3_Eu(PO_4_)_3_ prepared with a molar ratio of 3:0.5:3 was observed by TEM. The experimental results are shown in Figure 6.

From Figure 6a, it can be seen that the surface of Ba_3_Eu(PO_4_)_3_ was smooth, with some parts appearing light gray and the others appearing black. This indicated that the sample was composed of a combination of hollow and solid amorphous materials. Ba_3_Eu(PO_4_)_3_ particles prepared by the high-temperature solid-phase method become loose and expand at the outer edge due to the release of gas and heat. The local area (as shown in Figure 6b) was enlarged and the interplanar spacing between regions I and II was measured. The crystal plane spacing was 0.34 nm and 0.35 nm, respectively.

### 3.4. The Anti-Corrosion Performance of Ba_3_Eu(PO_4_)_3_ Coatings

#### 3.4.1. Salt Spray Resistance Test

**Figure 7:**
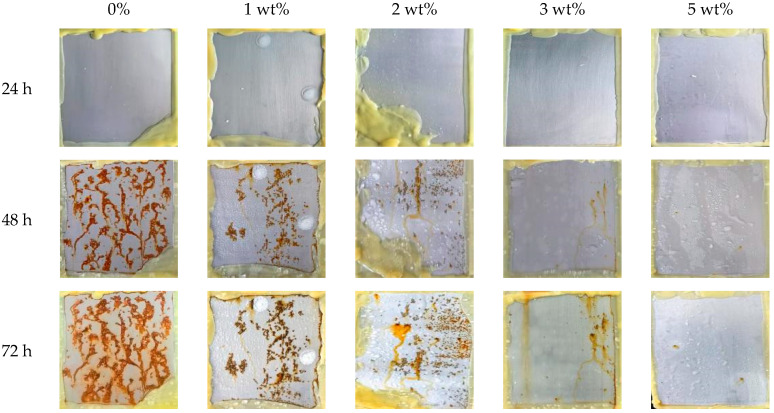
Salt spray resistance of coatings with different amounts of Ba_3_Eu(PO_4_)_3_.

Salt spray resistance is a corrosion testing method with accurate and practical reference value [18]. The salt spray resistance test was conducted on the coating without and with Ba_3_Eu(PO_4_)_3_ (the molar ratio was 3:0.5:3), and the results are shown in Figure 7.

Compared with the anti-corrosion effects of different coatings in Figure 7, it can be observed that, after exposure to neutral salt spray for 24 h, the coating with 1 wt% Ba_3_Eu(PO_4_)_3_ addition showed significant whitening and foaming. After 48 h, the pure acrylic resin coating corroded extensively, and the coatings with 1 wt% and 2 wt% Ba_3_Eu(PO_4_)_3_ showed filamentous corrosion. The coatings with 3 wt% and 5 wt% Ba_3_Eu(PO_4_)_3_ showed spotted rust spots. But the coating with 3 wt% Ba_3_Eu(PO_4_)_3_ had more rust spots than the one with 5 wt%. After 72 h, the corrosion area of the pure acrylic resin coating increased and granular rust spots appeared. As the amount of Ba_3_Eu(PO_4_)_3_ addition increases, a stable passivation film can be formed on the substrate, providing isolation and protection. Therefore, Ba_3_Eu(PO_4_)_3_ can effectively prevent corrosion of acrylic resin. The coating added with 5 wt% Ba_3_Eu(PO_4_)_3_ exhibited the highest corrosion resistance.

#### 3.4.2. SEM of Corrosion Coatings After Salt Spray Test

**Figure 8:**
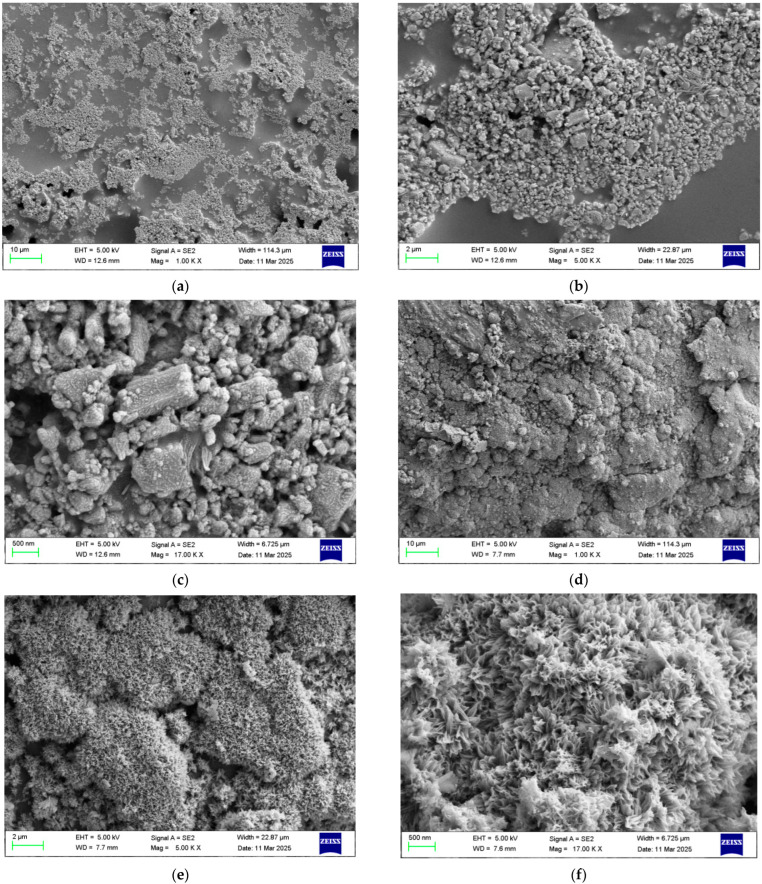
SEM of corrosion coatings after 72 h salt spray test: (**a**–**c**) pure acrylic resin coatings; (**d**–**f**) 5 wt% Ba_3_Eu(PO_4_)_3_ coatings.

The microstructure of pure acrylic and coatings containing 5 wt% Ba_3_Eu(PO_4_)_3_ after salt spray testing for 72 h is shown in Figure 8.

As shown in Figure 8a–c, pure acrylic resin was composed of cross-linked “dendritic” particle macromolecules, with elongated corrosion scales accumulating on the surface in an arc-shaped manner, exhibiting phenomena such as bubbling and cracking. From Figure 8d–f, it can be seen that the surface roughness of the acrylic coating containing Ba_3_Eu(PO_4_)_3_ increased. The distribution of corrosion products was uniform and presented a clustered “petal-like” shape. It indicated that a dense passivation film was formed on the surface of the coating sample with 5 wt% Ba_3_Eu(PO_4_)_3_ added, which could effectively block the shuttle of corrosive media.

#### 3.4.3. Electrochemical Test of Coatings

The Tafel curve is an important tool to reflect the kinetics of electrochemical reactions, providing a relationship between reaction rate and the concentration of reactants on the electrode surface. Electrochemical impedance spectroscopy (EIS) testing is a commonly used method for studying the corrosion resistance of coatings, which can intuitively reflect the corrosion behavior of coatings and help evaluate their long-term corrosion resistance [19]. By conducting immersion tests, impedance Bode plots, Nyquist plots, and phase plots were obtained to analyze and evaluate the anti-corrosion performance of the coating [20].

(1) Tafel Curve

**Figure 9:**
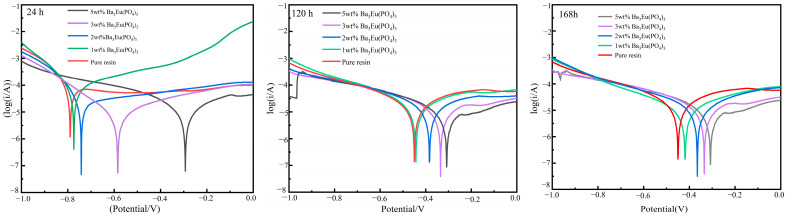
Tafel polarization curves of Ba_3_Eu(PO_4_)_3_ coatings with different added amounts over time.

The samples were soaked in a 3.5 wt% NaCl solution and the Tafel curves of Ba_3_Eu(PO_4_)_3_ coatings tested for 24 h, 120 h, and 168 h were made. The results are shown in Figure 9, and the specific test data are shown in Table 2.

From Figure 9 and Table 2, it was revealed that rare-earth phosphate as filler added to acrylic resin significantly changed the corrosion current density and corrosion voltage of the coating. The corrosion voltages of pure resin coating and coating with 1 wt% Ba_3_Eu(PO_4_)_3_ were not significantly different at 24 h, 120 h, and 168 h. At 24 h, the minimum corrosion current of the coating with 3 wt% Ba_3_Eu(PO_4_)_3_ was 7.793 μA/cm^2^, followed by the coating with 5 wt% Ba_3_Eu(PO_4_)_3_, which had a corrosion current of 8.82 μA/cm^2^. However, the 3 wt% E_corr_ (−0.676 V) was higher than 5 wt% E_corr_ (−0.736 V). That means the addition of 3 wt% coating had a better corrosion inhibition effect in the early stage of corrosion. At 120 h and 168 h, the corrosion current of the coating with 5 wt% Ba_3_Eu(PO_4_)_3_ is lower, being 10.96 μA/cm^2^ and 9.616 μA/cm^2^, respectively. The lower the corrosion current, the stronger the corrosion resistance of the material. When the corrosion currents of materials are not significantly different, those with higher corrosion potentials exhibit better corrosion resistance. The corrosion current and voltage of the coating with Ba_3_Eu(PO_4_)_3_ added were both lower than those of the pure acrylic resin, indicating that Ba_3_Eu(PO_4_)_3_ significantly enhanced the corrosion resistance of water-based acrylic coatings.

(2) Nyquist Curve

**Figure 10:**
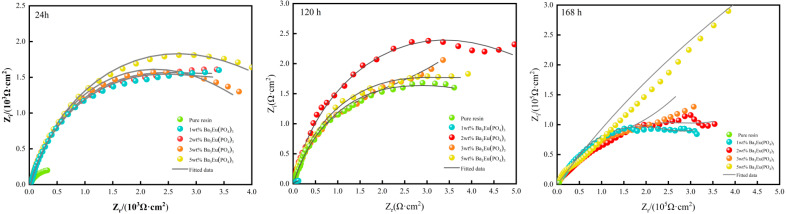
Nyquist plot of Ba_3_Eu(PO_4_)_3_ coating with different addition amounts over time.

Coatings with different amounts of Ba_3_Eu(PO_4_)_3_ were immersed in 3.5 wt% NaCl aqueous solution for 24 h, 120 h, and 168 h, respectively. The experimental results were fitted and analyzed using an equivalent circuit model. Subsequently, the electrochemical parameters of the coating corrosion process were displayed as Nyquist plots, shown in Figure 10.

The arc radius of the Nyquist plot represents the impedance value. The larger the arc radius, the better the anti-corrosion performance of the coating [21]. From Figure 9, it can be seen that the capacitance arc diameter of the coating with added Ba_3_Eu(PO_4_)_3_ was always larger than that of pure acrylic coating. As the soaking time increased, the capacitance arc radius decreased, which may be due to the infiltration of corrosive media into the coating. The resistance R of the coating gradually decreased, leading to a decrease in anti-corrosion effect. However, the capacitance arc diameter of the 5 wt% Ba_3_Eu(PO_4_)_3_ coating was the largest.

(3) Bode Curve

**Figure 11:**
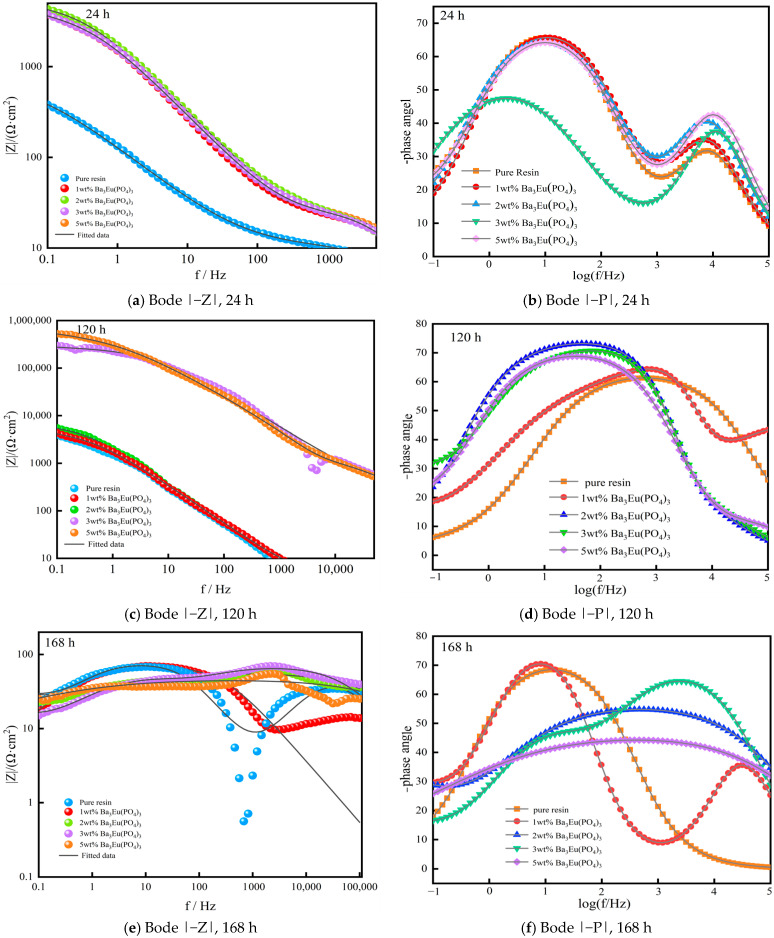
Bode plots of Ba_3_Eu(PO_4_)_3_ coating with different addition amounts over time.

Figure 11 shows the Bode plots of coatings with different amounts of Ba_3_Eu(PO_4_)_3_ addition and immersed in 3.5 wt% NaCl solution for 24 h, 120 h, and 168 h.

In the Bode plot of impedance modulus values, the time constant in the mid- to high-frequency range represents the characteristics of the coating. Meanwhile, the impedance modulus values below the mid- to low-frequency range (f < 0.1 Hz) are frequency independent and mainly used to measure the protective performance of the coating. The larger the value, the better the protective effect of the coating [22]. The time constant in the low-frequency range represents the characteristics of metal interface corrosion under the coating. In the Bode phase angle curve, the higher the phase angle curve, the smaller the coating capacitance value, the larger the resistance value, and the less electrolyte infiltration into the coating/metal interface [23]. In addition, in the phase angle curve, a peak–valley pair represents a time constant, which can be used to determine the corrosion process of the coating system with the Nyquist plot [24].

From the Bode plots in Figure 11b,d,f, it can be seen that the coating system exhibited at least one time constant. Combined with the Nyquist plot, it was believed that the coating system had two time constants, namely, the impedance spectrum of the coating showed two capacitive arcs. Coatings with different Ba_3_Eu(PO_4_)_3_ addition amounts exhibited different amplitudes of capacitive arcs in the high-frequency range. Overall, the addition of 2 wt% and 5 wt% yielded better results. From the Bode plots in Figure 11a,c,e, the 3 wt% Ba_3_Eu(PO_4_)_3_ coating had the highest impedance in the low-frequency region after 24 h and 120 h of corrosion. However, the impedance value of the 5 wt% addition was significantly lower compared to the low-frequency region, while the impedance value of the 2–5 wt% additions was similar in the high-frequency region. This may be due to the saturation of the coating, resulting in a decrease in the anti-corrosion performance of the coating [25,26]. By 168 h, the Ba_3_Eu(PO_4_)_3_ coating containing 2 wt% and 5 wt% exhibited high impedance in both low- and high-frequency regions.

#### 3.4.4. Equivalent Circuit

**Figure 12:**
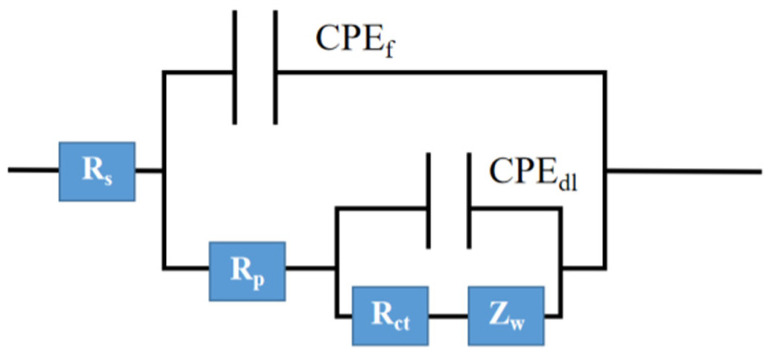
The Equivalent circuit of coatings.

The equivalent circuit diagram and data according to the Nyquist curve are shown in Figure 12 and Table 3.

R_s_, R_f_, and R_ct_ represent solution resistance, membrane resistance, and charge transfer resistance, respectively. CPE_f_, CPE_dl_, and Z_w_ are thin film capacitors, double-layer capacitors, and Warburg diffusion coefficients, respectively [27].

The corrosion rate of metals decreases with the increase in R_ct_ and R_p_. In the later stage of corrosion, the electrolyte is closely related to the dielectric constant, and an increase in CPE_f_ promotes the diffusion of the electrolyte [28]. The R_ct_ value is positively correlated with the protective performance of the coating, while a decrease indicates a decrease in the resistance of the coating to the penetration of corrosive media [29].

From Table 3, it can be seen that the R_ct_ values of Ba_3_Eu(PO_4_)_3_ coatings with addition amounts of 2 wt%, 3 wt%, and 5 wt% showed an overall upward trend, indicating that the Ba_3_Eu(PO_4_)_3_ deposited on the substrate surface can effectively suppress grain boundary corrosion after the corrosive medium enters the coating. Ba_3_Eu(PO_4_)_3_ slowly released Eu^3+^ ions and reacted with hydroxyl ions, which were generated in the cathode region to form a protective layer. Phosphates as corrosion inhibitors formed a dense protective film on the surface of the substrate, which can achieve a dual anti-corrosion effect. In summary, the type and amount of fillers are closely related to the anti-corrosion performance of the coating.

### 3.5. Anti-Corrosion Mechanism

**Figure 13:**
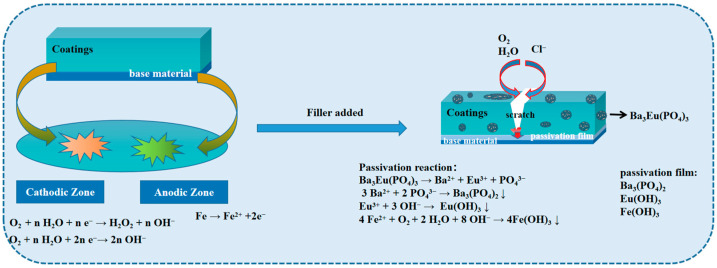
Schematic diagram of corrosion protection mechanism of Ba_3_Eu(PO_4_)_3_.

The anti-corrosion mechanism of Ba_3_Eu(PO_4_)_3_ in the presence of an acrylic coating is shown in Figure 13.

There are usually four reasons for corrosion, namely, physical corrosion, chemical corrosion, electrochemical corrosion, and microbial corrosion. Due to the presence of micropores or defects on the surface of metal substrates, primary batteries are easily formed, where the anode region substrate loses electrons and becomes Fe^2+^ and the cathode region produces OH^−^. Therefore, this system is mainly characterized by electrochemical corrosion. The corrosion rate depends on the ion migration rate, ion concentration, and the difference in positive and negative electrode potentials.

With the penetration of corrosive media, the acrylic coating containing Ba_3_Eu(PO_4_)_3_ ionizes to form Ba^2+^, Eu^3+^, and PO_4_^3−^, which first form Ba_3_(PO_4_)_3_ precipitate (k_sp_ = 3.4 × 10^−23^), effectively reducing the ion concentration of the microcell. Eu^3+^ plasma has a larger radius, slower migration speed, and lower corrosion current. In an alkaline system, Eu^3+^ and Fe^3+^ were deposited and passivated, resulting in Eu(OH)_3_ and Fe(OH)_3_ precipitates [30] (k_sp_ was 8.9 × 10^−24^ and 4.0 × 10^−38^, respectively), effectively reducing the potential difference between the positive and negative electrodes and slowing down the electrochemical corrosion rate. Secondly, the passivation film was dense and highly amorphous, which had a certain barrier effect on oxygen. Therefore, as the amount of Ba_3_Eu(PO_4_)_3_ addition increases, the thickness of the passivation film increased and the anti-corrosion effect became more excellent. According to Table 2, the increase in corrosion potential impedes the anodic reaction. Table 3 shows that, as the charge transfer resistance increases, the hindrance to charge transfer between the electrode and the corrosive solution also increases [31]. The results of the salt spray resistance test and electrochemical test in Figure 10 were consistent. The correctness of the anti-corrosion mechanism diagram of the acrylic coating with added Ba_3_Eu(PO_4_)_3_ was verified.

## 4. Conclusions

In this study, a novel Ba_3_Eu(PO_4_)_3_ as white powder and with a fluffy pore structure was prepared by a high-temperature solid-phase method. When the mole ratio of BaCO_3_:Eu_2_O_3_:(NH_4_)H_2_PO_4_ = 3:0.5:3 and it is ground for 30 min and calcined at 950 °C for 2000 min, the purest product was obtained. Compared with pure resin, the salt spray resistance test showed that the coating filled with Ba_3_Eu(PO_4_)_3_ had excellent barrier properties. When the addition amount of Ba_3_Eu(PO_4_)_3_ was 5 wt%, the anti-corrosion effect was the highest. The corrosion current was minimal at 9.616 μA/cm^2^. A mechanism model for adding Ba_3_Eu(PO_4_)_3_ anti-corrosion coating was established.

## Figures and Tables

**Table 1:** Formula of rare-earth barium phosphate coatings.

Raw Material	wt/%
Waterborne acrylic lotion	50.0–60.0
Defoamer C-15	0.4–0.6
Deionized water	15.0–8.0
pH regulator DMAE	0.2–0.4
Wetting agent G-033	0.5–1.0
Leveling agent HTK-3020	0.5–0.8
Film-forming aid alcohol ester 12	2.4–3.0
Anti-flash-rust agent NaNO_2_	1.0–2.0
Thickener WT-105A	appropriate amount
Dispersant 5040	0.4–0.8
Ba_3_Eu(PO_4_)_3_	0–5.0
Total	100

**Table 2:** Tafel test data of coatings with different Ba_3_Eu(PO_4_)_3_ additions.

Samples	Time (h)	E_corr_ (V)	I_corr_ (μA/cm^2^)	R_p_ (Ω)	b_a_	−b_c_
0 wt%	24	−1.068	135.90	372	0.817	7.785
	120	−1.127	505.20	107	1.309	6.754
	168	−1.082	495.70	122	0.076	7.104
1 wt%	24	−1.036	80.62	447	3.441	8.617
	120	−1.028	76.92	567	1.476	8.497
	168	−1.026	70.65	3419	3.156	5.108
2 wt%	24	−1.015	24.03	1232	1.736	8.866
	120	−0.888	14.70	2977	4.652	5.285
	168	−0.784	12.92	3416	4.357	5.493
3 wt%	24	−0.676	7.793	5202	4.321	6.404
	120	−0.916	15.39	3419	3.156	5.108
	168	−0.868	13.33	3300	4.604	5.281
5 wt%	24	−0.736	8.82	4591	4.504	6.232
	120	−0.794	10.96	3734	5.521	5.107
	168	−0.838	9.616	5023	3.714	5.288

E_corr_: corrosion potential, V; I_corr_: corrosion current density, μA/cm^2^; R_p_: polarization resistance; b_a_: the anode Tafel slope; b_c_: the cathode Tafel slope.

**Table 3:** The electrochemical impedance spectroscopy data of coatings with different Ba_3_Eu(PO_4_)_3_ additions.

Time (h)	Additive Amount (wt%)	R_s_ (Ω·cm^2^)	CPE_f_ (Ω^−1^·cm^−2^·s^−n^)	R_p_ (Ω·cm ^2^)	CPE_dl_ (Ω^−1^·cm^−2^·s^−n^)	R_ct_ (Ω·cm^2^)	Z_w_ (Ω·cm^2^)
24	0	6.27	7.18 × 10^−6^	193.70	1.14 × 10^−4^	3499	0.002
	1	4.96	5.05 × 10^−6^	22.31	1.1 × 10^−4^	3840	0.030
	2	4.49	6.34 × 10^−6^	23.50	1.1 × 10^−4^	4766	0.002
	3	3.79	4.95 × 10^−6^	22.07	1.2 × 10^−4^	3654	0.002
	5	5.45	7.58 × 10^−6^	20.64	9.85 × 10^−5^	4112	0.030
120	0	0.01	3.59 × 10^−6^	341.30	2.46 × 10^−7^	1.58 × 10^9^	2.90 × 10^−7^
	1	36.75	6.08 × 10^−7^	2058	1.38 × 10^−8^	5.37 × 10^5^	9.66 × 10^−6^
	2	2.70	8.62 × 10^−5^	3.03	1.04 × 10^−4^	7.83 × 10^6^	1.31 × 10^−3^
	3	2.51	1.08 × 10^−4^	3.42	8.41 × 10^−6^	3160	5.99 × 10^−4^
	5	2.90	4.72 × 10^−5^	2.10	6.02 × 10^−6^	4154	1.05 × 10^−3^
168	0	17.56	8.64 × 10^−5^	5012	3.50 × 10^−3^	533	3.69 × 10^7^
	1	11.36	7.95 × 10^−5^	42.77	5.73 × 10^−5^	3071	4.77 × 10^−4^
	2	122.40	1.16 × 10^−6^	23,600	8.42 × 10^−8^	1.79 × 10^3^	6.76 × 10^−6^
	3	183.80	1.70 × 10^−7^	3180	7.11 × 10^−7^	2.73 × 10^3^	1.63 × 10^−6^
	5	9192	4.38 × 10^−11^	6567	3.28 × 10^−8^	4.03 × 10^7^	3.81 × 10^−8^

## Data Availability

The data presented in this study are available in the article.
